# Factors Associated With US Public Motivation to Use and Distribute COVID-19 Self-tests

**DOI:** 10.1001/jamanetworkopen.2020.34001

**Published:** 2021-01-20

**Authors:** Cedric Bien-Gund, Karen Dugosh, Trisha Acri, Kathleen Brady, Harsha Thirumurthy, Jessica Fishman, Robert Gross

**Affiliations:** 1 Division of Infectious Diseases, University of Pennsylvania Perelman School of Medicine, University of Pennsylvania, Philadelphia; 2 Public Health Management Corporation, Philadelphia, Pennsylvania; 3 Philadelphia Department of Public Health, AIDS Activities Coordinating Office, Philadelphia, Pennsylvania; 4 Division of Health Policy, University of Pennsylvania Perelman School of Medicine, Philadelphia; 5 Department of Psychiatry, University of Pennsylvania Perelman School of Medicine, Philadelphia

## Abstract

This survey study examines factors associated with motivation to use and distribute self-tests for COVID-19 infection among US adults.

## Introduction

The coronavirus disease 2019 (COVID-19) pandemic has compelled the rapid development and implementation of novel diagnostics to detect the causative virus, severe acute respiratory syndrome coronavirus 2 (SARS-CoV-2). One potential strategy to increase testing coverage is the secondary distribution of COVID-19 self-testing kits,^1,2^ whereby at-risk or infected individuals distribute test kits to contacts in their social network; this strategy has been successfully implemented to increase HIV testing.^3^ Furthermore, this strategy may be associated with improved contact tracing by facilitating case detection if test kits are distributed to contacts of infected individuals. Given the urgent need to increase COVID-19 testing coverage and contact tracing, we examined individuals’ motivation to self-test and to distribute self-test kits.

## Methods

This survey study used data from Amazon’s Mechanical Turk (MTurk), an online platform that provides high-quality health-related data.^4^ We limited participation to US adults 18 years or older. The University of Pennsylvania institutional review board reviewed and approved this study and granted a waiver of informed consent because the research presented no more than minimal risk of harm to participants and involved no procedures for which informed consent is normally required. This study followed American Association for Public Opinion Research (AAPOR) reporting guideline.

Participants were given information about self-test kits and an explanation of how the kits were to be used. We used 6-point scales (1 indicating extremely unlikely and 6 indicating extremely likely) to measure levels of motivation to do the following: (1) distribute self-test kits to contacts if the individual tested positive, (2) self-test if the individual received a kit from a potentially infected contact, and (3) order a free self-test kit online if exposed to COVID-19. We dichotomized levels of motivation (1-3 vs 4-6) and used logistic regression to assess the association between motivation and specific sociodemographic characteristics (age, income, gender, race/ethnicity, marital status, and education). All tests were 2-sided, and statistical significance was set at *P* < .05. Analyses were performed using Stata, version 15.1 (StataCorp LLC).

## Results

In a sample of 586 adults, 350 (59.7%) were male and the median age was 35 years (interquartile range [IQR], 29-42 years) (Table). Motivation to perform each of the self-testing activities was high: 526 of 584 respondents (90.1%) were motivated to distribute self-testing kits to contacts (median score, 5; IQR, 4-6), 503 of 584 (86.1%) were motivated to self-test if given a kit from a potentially infected contact (median score, 5; IQR, 4-5), and 482 of 582 (82.8%) were motivated to order a self-test kit online (median score, 5; IQR, 4-5).

**Table.** Characteristics of US Adults Willing to Distribute COVID-19 Self-tests and to Perform Self-testing

Characteristic	Total sample, No. (%) (N = 586)	Motivation to distribute self-test kits if infected with COVID-19 (n = 526)^a^				Motivation to self-test if kit received from a potentially infected contact (n = 503)^a^				Motivation to order self-test kits online if exposed to COVID-19 (n = 482)^a^			
		No. (%)	aOR (95% CI)	*P* value	No. (%)		aOR (95% CI)	*P* value	No. (%)		aOR (95% CI)	*P* value
Age, median (IQR), y	35 (29-42)	35 (28-42)	0.99 (0.97-1.02)	.54	35 (28-42)		0.99 (0.97-1.01)	.47	34 (28-43)		1.00 (0.98-1.03)	.72
Gender												
Male	350 (59.7)	317 (60.3)	1 [Reference]	NA	301 (59.8)		1 [Reference]	NA	287 (59.5)		1 [Reference]	NA
Female	234 (39.9)	208 (39.5)	1.00 (0.56-1.81)	.99	201 (40.0)		1.17 (0.70-1.94)	.55	195 (40.5)		1.21 (0.75-1.96)	.43
Nonbinary	2 (0.3)	1 (0.2)	0.15 (0.01-3.84)	.25	1 (0.2)		0.19 (0.01-3.77)	.28	0		NA	NA
Race/ethnicity												
Non-Hispanic White	293 (50.0)	258 (49.0)	1 [Reference]	NA	236 (46.9)		1 [Reference]	NA	230 (47.7)		1 [Reference]	NA
Non-Hispanic Black	67 (11.4)	60 (11.4)	0.87 (0.35-2.15)	.76	59 (11.7)		1.53 (0.67-3.52)	.31	58 (12.0)		1.38 (0.63-3.06)	.42
Hispanic	196 (33.4)	181 (34.4)	1.49 (0.72-3.10)	.29	179 (35.6)		2.36 (1.25-4.44)	.008	173 (35.9)		1.93 (1.07-3.48)	.03
Non-Hispanic other	30 (5.1)	27 (5.1)	1.16 (0.32-4.21)	.82	29 (5.8)		6.98 (0.92-53.12)	.06	21 (4.4)		0.57 (0.24-1.38)	.21
Marital status												
Not married	135 (23.0)	113 (21.5)	1 [Reference]	NA	108 (21.5)		1 [Reference]	NA	101 (21.0)		1 [Reference]	NA
Married	451 (77.0)	413 (78.5)	1.12 (0.53-2.35)	.77	395 (78.5)		0.96 (0.50-1.83)	.94	381 (79.0)		0.91 (0.49-1.68)	.76
Income^b^												
Below average	80 (13.7)	62 (11.8)	1 [Reference]	NA	58 (11.5)		1 [Reference]	NA	53 (11.0)		1 [Reference]	NA
Average	219 (37.4)	196 (37.3)	1.83 (0.80-4.18)	.15	178 (35.4)		1.35 (0.66-2.73)	.41	168 (34.9)		1.59 (0.81-3.11)	.18
Above average	287 (49.0)	268 (51.0)	3.04 (1.26-7.32)	.01	267 (53.1)		4.15 (1.87-9.25)	<.001	261 (54.1)		4.77 (2.25-10.10)	<.001
Education												
High school or less	21 (3.6)	15 (2.9)	1 [Reference]	NA	17 (3.4)		1 [Reference]	NA	18 (3.7)		1 [Reference]	NA
Some college	79 (13.5)	61 (11.6)	1.08 (0.33-3.52)	.89	61 (12.1)		0.82 (0.23-2.95)	.76	51 (10.6)		0.27 (0.07-1.05)	.06
Completed 4-y college	419 (71.5)	389 (74.0)	3.43 (1.12-10.50)	.03	367 (73.0)		1.28 (0.39-4.23)	.68	357 (74.1)		0.71 (0.20-2.62)	.61
>4-y College	67 (11.4)	61 (11.6)	2.45 (0.63-9.49)	.20	58 (11.5)		0.93 (0.24-3.67)	.92	56 (11.6)		0.50 (0.12-2.11)	.35

Abbreviations: aOR, adjusted odds ratio; COVID-19, coronavirus disease 2019; IQR, interquartile range; NA, not applicable.

^a^ Proportion reporting motivation calculated excluding those with missing responses.

^b^ Income is self-reported, and individuals were asked to define their income as below average, average, or above average without specific dollar amounts.

In multivariable analyses, motivation to distribute self-test kits was associated with above-average income (vs below-average income: adjusted odds ratio [aOR], 3.04; 95% CI, 1.26-7.32) and completion of college (vs high school or less: aOR, 3.43; 95% CI, 1.12-10.50). Both motivation to self-test after receiving a test kit from a potentially infected contact and motivation to order a self-test kit online were associated with above-average income (vs below-average income: aOR, 4.15 [95% CI, 1.87-9.25] and 4.7 [95% CI, 2.25-10.10], respectively) and Hispanic ethnicity (vs non-Hispanic White ethnicity; aOR, 2.36 [95% CI, 1.25-4.44] and 1.93 [95% CI, 1.07-3.48], respectively).

## Discussion

We found that the majority of survey participants were motivated to distribute COVID-19 self-test kits and to use self-test kits. Motivation is a prerequisite for voluntary behavior,^5^ and our findings suggest that the secondary distribution of COVID-19 self-test kits may be associated with increased test uptake and case detection. However, individuals with lower socioeconomic status reported lower motivation and may be less likely to distribute test kits and self-test; behavioral interventions may help increase motivation in this population. Limitations include use of online sampling methods, which may limit generalizability of prevalence estimates and introduce sampling bias; however, observed associations from MTurk sampling are often comparable to those obtained by conventional survey methods.^4^ In addition, we did not assess behavioral outcomes, and social desirability may be present with self-report data. Nonetheless, measures of motivation have been shown to more accurately predict health behavior than alternative variables.^5^ Future research may help determine whether COVID-19 self-testing and secondary distribution of self-test kits are associated with increased testing coverage.

## References

[1] McCulloch DJ, Kim AE, Wilcox NC, Comparison of unsupervised home self-collected midnasal swabs with clinician-collected nasopharyngeal swabs for detection of SARS-CoV-2 infection. JAMA Netw Open. 2020;3(7):e2016382-e2016382. doi:10.1001/jamanetworkopen.2020.16382 32697321 PMC7376392

[2] Altamirano J, Govindarajan P, Blomkalns AL, Assessment of sensitivity and specificity of patient-collected lower nasal specimens for sudden acute respiratory syndrome coronavirus 2 testing. JAMA Netw Open. 2020;3(6):e2012005-e2012005. doi:10.1001/jamanetworkopen.2020.12005 32530469 PMC7292998

[3] Masters SH, Agot K, Obonyo B, Napierala Mavedzenge S, Maman S, Thirumurthy H Promoting partner testing and couples testing through secondary distribution of HIV self-tests: a randomized clinical trial. PLoS Med. 2016;13(11):e1002166. doi:10.1371/journal.pmed.1002166 27824882 PMC5100966

[4] Mortensen K, Hughes TL Comparing Amazon’s Mechanical Turk platform to conventional data collection methods in the health and medical research literature. J Gen Intern Med. 2018;33(4):533-538. doi:10.1007/s11606-017-4246-0 29302882 PMC5880761

[5] Fishman J, Lushin V, Mandell DS Predicting implementation: comparing validated measures of intention and assessing the role of motivation when designing behavioral interventions. Implement Sci Commun. 2020;1:81. doi:10.1186/s43058-020-00050-4 33005900 PMC7523324

